# Supplementary material for: Quantifying individual differences in brain morphometry underlying symptom severity in Autism Spectrum Disorders
Source: Sci Rep. 2019 Jul 9;9:9898. doi: 10.1038/s41598-019-45774-z (PMC6617442; doi:10.1038/s41598-019-45774-z)
Supplement: Supplementary file 1 — Supplementary material [file 41598_2019_45774_MOESM1_ESM.docx]

**Quantifying individual differences in brain morphometry underlying symptom severity in Autism Spectrum Disorders**

Emmanuel Peng Kiat Pua^1,2^, Gareth Ball^2^, Chris Adamson^2^, Stephen Bowden^1,4^, and Marc L Seal^2,3^

Author Affiliations

^1^ Melbourne School of Psychological Sciences, University of Melbourne, Australia

^2^ Developmental Imaging, Murdoch Children’s Research Institute, Australia

^3^ Department of Paediatrics, University of Melbourne, Australia

^4^ St. Vincent’s Hospital, Melbourne, Australia

Corresponding Author

Emmanuel Pua

Developmental Imaging, Murdoch Children’s Research Institute

50 Flemington Rd, The Royal Children’s Hospital, Parkville, VIC 3052, Melbourne, Australia

+61 (03) 99366420 [emmanuel.pua@mcri.edu.au](mailto:emmanuel.pua@mcri.edu.au)

# Supplementary material

## Supplementary Table 1. Mean beta coefficients of cortical thickness features selected by regularized regression with elastic net penalty

| Cortical thickness features | Hemisphere | Mean | SD | Lower bound | Upper bound |
| --- | --- | --- | --- | --- | --- |
| Inferior temporal gyrus | R | 0.49 | 0.022 | 0.45 | 0.54 |
| Pars orbitalis | L | 0.26 | 0.0058 | 0.25 | 0.27 |
| Cuneus cortex | R | 0.23 | 0.0059 | 0.22 | 0.24 |
| Inferior parietal lobule | R | 0.19 | 0.012 | 0.16 | 0.21 |
| Insula | R | 0.15 | 0.0090 | 0.13 | 0.17 |
| Isthmus cingulate gyrus | R | 0.15 | 0.0066 | 0.14 | 0.17 |
| Medial orbitofrontal | R | 0.11 | 0.0050 | 0.10 | 0.12 |
| Posterior cingulate gyrus | R | 0.10 | 0.0021 | 0.099 | 0.11 |
| Caudal anterior cingulate gyrus | R | 0.098 | 0.0052 | 0.089 | 0.11 |
| Rostral middle frontal gyrus | L | 0.00069 | 0.0022 | 0.00 | 0.0093 |
| Pericalcarine cortex | R | -0.049 | 0.0037 | -0.057 | -0.042 |
| Pars triangularis | R | -0.052 | 0.0015 | -0.055 | -0.049 |
| Fusiform gyrus | L | -0.062 | 0.0029 | -0.068 | -0.056 |
| Middle temporal gyrus | L | -0.082 | 0.0014 | -0.085 | -0.080 |
| Lingual gyrus | L | -0.14 | 0.0066 | -0.15 | -0.13 |
| Entorhinal cortex | R | -0.15 | 0.0083 | -0.17 | -0.13 |
| Inferior temporal gyrus | L | -0.21 | 0.014 | -0.24 | -0.19 |
| Fusiform gyrus | R | -0.24 | 0.0039 | -0.25 | -0.23 |
| Middle temporal gyrus | R | -0.36 | 0.018 | -0.40 | -0.33 |

## Supplementary Table 2. Mean beta coefficients of surface area features selected by regularized regression with elastic net penalty

| Surface area features | Hemisphere | Mean | SD | Lower bound | Upper bound |
| --- | --- | --- | --- | --- | --- |
| Isthmus cingulate gyrus | L | 0.23 | 0.0079 | 0.21 | 0.24 |
| Caudal middle frontal gyrus | L | 0.15 | 0.0063 | 0.13 | 0.15 |
| Supramarginal gyrus | L | 0.13 | 0.0038 | 0.12 | 0.13 |
| Entorhinal cortex | R | 0.11 | 0.0028 | 0.11 | 0.16 |
| Isthmus cingulate gyrus | R | -0.14 | 0.0055 | -0.15 | -0.13 |
| Rostral anterior cingulate gyrus | R | -0.15 | 0.00012 | -0.15 | -0.15 |
| Rostral anterior cingulate gyrus | L | -0.15 | 0.0029 | -0.15 | -0.14 |
| Inferior parietal lobule | L | -0.15 | 0.0057 | -0.16 | -0.14 |
| Rostral middle frontal gyrus | L | -0.21 | 0.0069 | -0.21 | -0.19 |
| Insula | L | -0.24 | 0.0052 | -0.24 | -0.23 |

## Supplementary Table 3. Comparison of model fit in subject-level, distance-based model to the group-level model

| **Model** | **R^2^** | **Mean Squared Error** | **Correlation, *r*** |
| --- | --- | --- | --- |
| **Cortical Thickness** |  |  |  |
| Subject-level model | 0.15 | 0.98 | 0.39 |
| Group-level model | 0.0000434 | 1.025 | -0.00659 |
| **Surface Area** |  |  |  |
| Subject-level model | 0.18 | 1.01 | 0.42 |
| Group-level model | 0.0158 | 1.009 | 0.13 |

*Note. Cortical features selected by our approach (subject-level, distance-based model) significantly outperformed common methods (group-level modelling) in predicting symptom severity in independent out-of-sample validation testing. Both models were trained and evaluated using the same rigorous machine learning pipeline.*

## Supplementary Table 4. Matched subject pairs from ABIDE-II

| ABIDE-II ID | PAIR ID | Diagnosis |
| --- | --- | --- |
| 29082 | 1 | 0 |
| 29078 | 2 | 0 |
| 29073 | 4 | 0 |
| 28762 | 6 | 0 |
| 28798 | 7 | 0 |
| 28837 | 9 | 0 |
| 28748 | 10 | 0 |
| 28743 | 11 | 0 |
| 28847 | 12 | 0 |
| 28826 | 13 | 0 |
| 28842 | 15 | 0 |
| 28812 | 16 | 0 |
| 28785 | 17 | 0 |
| 28767 | 18 | 0 |
| 28830 | 19 | 0 |
| 28793 | 22 | 0 |
| 28804 | 23 | 0 |
| 28836 | 24 | 0 |
| 28806 | 25 | 0 |
| 28774 | 26 | 0 |
| 28813 | 27 | 0 |
| 29418 | 34 | 0 |
| 29359 | 36 | 0 |
| 29444 | 37 | 0 |
| 29457 | 38 | 0 |
| 29303 | 39 | 0 |
| 29402 | 40 | 0 |
| 29334 | 42 | 0 |
| 29429 | 43 | 0 |
| 29364 | 44 | 0 |
| 29379 | 45 | 0 |
| 29309 | 46 | 0 |
| 29431 | 47 | 0 |
| 29356 | 48 | 0 |
| 29336 | 50 | 0 |
| 29337 | 51 | 0 |
| 29399 | 52 | 0 |
| 29346 | 53 | 0 |
| 29455 | 54 | 0 |
| 29424 | 55 | 0 |
| 29460 | 56 | 0 |
| 29388 | 57 | 0 |
| 29323 | 58 | 0 |
| 29305 | 59 | 0 |
| 29328 | 60 | 0 |
| 29378 | 61 | 0 |
| 29299 | 62 | 0 |
| 29422 | 63 | 0 |
| 29395 | 64 | 0 |
| 29318 | 65 | 0 |
| 29470 | 66 | 0 |
| 29446 | 68 | 0 |
| 29369 | 69 | 0 |
| 29325 | 70 | 0 |
| 29316 | 71 | 0 |
| 29427 | 72 | 0 |
| 29301 | 73 | 0 |
| 29238 | 74 | 0 |
| 29254 | 76 | 0 |
| 29244 | 77 | 0 |
| 29232 | 78 | 0 |
| 29226 | 79 | 0 |
| 29253 | 80 | 0 |
| 29228 | 81 | 0 |
| 29252 | 82 | 0 |
| 29250 | 83 | 0 |
| 29241 | 84 | 0 |
| 29239 | 85 | 0 |
| 29236 | 86 | 0 |
| 29234 | 87 | 0 |
| 29227 | 88 | 0 |
| 29229 | 89 | 0 |
| 29237 | 90 | 0 |
| 29230 | 91 | 0 |
| 29235 | 92 | 0 |
| 28862 | 93 | 0 |
| 28858 | 94 | 0 |
| 28878 | 95 | 0 |
| 28867 | 96 | 0 |
| 28893 | 97 | 0 |
| 28888 | 98 | 0 |
| 28863 | 99 | 0 |
| 28889 | 100 | 0 |
| 28892 | 101 | 0 |
| 28900 | 102 | 0 |
| 28870 | 103 | 0 |
| 28881 | 104 | 0 |
| 28895 | 105 | 0 |
| 29134 | 118 | 0 |
| 29135 | 119 | 0 |
| 29117 | 120 | 0 |
| 29124 | 121 | 0 |
| 29132 | 122 | 0 |
| 29128 | 123 | 0 |
| 29130 | 124 | 0 |
| 30007 | 125 | 0 |
| 30004 | 127 | 0 |
| 30029 | 128 | 0 |
| 30024 | 129 | 0 |
| 30015 | 130 | 0 |
| 29058 | 1 | 1 |
| 29059 | 2 | 1 |
| 29064 | 4 | 1 |
| 28752 | 6 | 1 |
| 28761 | 7 | 1 |
| 28765 | 9 | 1 |
| 28771 | 10 | 1 |
| 28777 | 11 | 1 |
| 28778 | 12 | 1 |
| 28779 | 13 | 1 |
| 28784 | 15 | 1 |
| 28790 | 16 | 1 |
| 28796 | 17 | 1 |
| 28800 | 18 | 1 |
| 28815 | 19 | 1 |
| 28819 | 22 | 1 |
| 28821 | 23 | 1 |
| 28834 | 24 | 1 |
| 28835 | 25 | 1 |
| 28838 | 26 | 1 |
| 28839 | 27 | 1 |
| 29273 | 34 | 1 |
| 29277 | 36 | 1 |
| 29278 | 37 | 1 |
| 29279 | 38 | 1 |
| 29280 | 39 | 1 |
| 29283 | 40 | 1 |
| 29285 | 42 | 1 |
| 29286 | 43 | 1 |
| 29287 | 44 | 1 |
| 29288 | 45 | 1 |
| 29289 | 46 | 1 |
| 29290 | 47 | 1 |
| 29291 | 48 | 1 |
| 29344 | 50 | 1 |
| 29375 | 51 | 1 |
| 29376 | 52 | 1 |
| 29385 | 53 | 1 |
| 29389 | 54 | 1 |
| 29391 | 55 | 1 |
| 29392 | 56 | 1 |
| 29393 | 57 | 1 |
| 29394 | 58 | 1 |
| 29401 | 59 | 1 |
| 29404 | 60 | 1 |
| 29408 | 61 | 1 |
| 29411 | 62 | 1 |
| 29412 | 63 | 1 |
| 29413 | 64 | 1 |
| 29416 | 65 | 1 |
| 29417 | 66 | 1 |
| 29433 | 68 | 1 |
| 29434 | 69 | 1 |
| 29435 | 70 | 1 |
| 29449 | 71 | 1 |
| 29458 | 72 | 1 |
| 29477 | 73 | 1 |
| 29179 | 74 | 1 |
| 29186 | 76 | 1 |
| 29188 | 77 | 1 |
| 29193 | 78 | 1 |
| 29194 | 79 | 1 |
| 29195 | 80 | 1 |
| 29196 | 81 | 1 |
| 29199 | 82 | 1 |
| 29200 | 83 | 1 |
| 29201 | 84 | 1 |
| 29204 | 85 | 1 |
| 29206 | 86 | 1 |
| 29207 | 87 | 1 |
| 29208 | 88 | 1 |
| 29217 | 89 | 1 |
| 29218 | 90 | 1 |
| 29152 | 91 | 1 |
| 29157 | 92 | 1 |
| 28857 | 93 | 1 |
| 28860 | 94 | 1 |
| 28864 | 95 | 1 |
| 28865 | 96 | 1 |
| 28866 | 97 | 1 |
| 28874 | 98 | 1 |
| 28875 | 99 | 1 |
| 28890 | 100 | 1 |
| 28897 | 101 | 1 |
| 28898 | 102 | 1 |
| 28899 | 103 | 1 |
| 28905 | 104 | 1 |
| 28908 | 105 | 1 |
| 29098 | 118 | 1 |
| 29104 | 119 | 1 |
| 29105 | 120 | 1 |
| 29108 | 121 | 1 |
| 29109 | 122 | 1 |
| 29114 | 123 | 1 |
| 29115 | 124 | 1 |
| 30000 | 125 | 1 |
| 30014 | 127 | 1 |
| 30017 | 128 | 1 |
| 30019 | 129 | 1 |
| 30027 | 130 | 1 |

*Note.* ABIDE-II ID: ABIDE-II subject identifier.

Pair ID: Study assigned identifier for matched pairs;

DX_Group: ABIDE-II diagnosis labels. 0 for Controls, 1 for ASD.

## Supplementary Figure 1


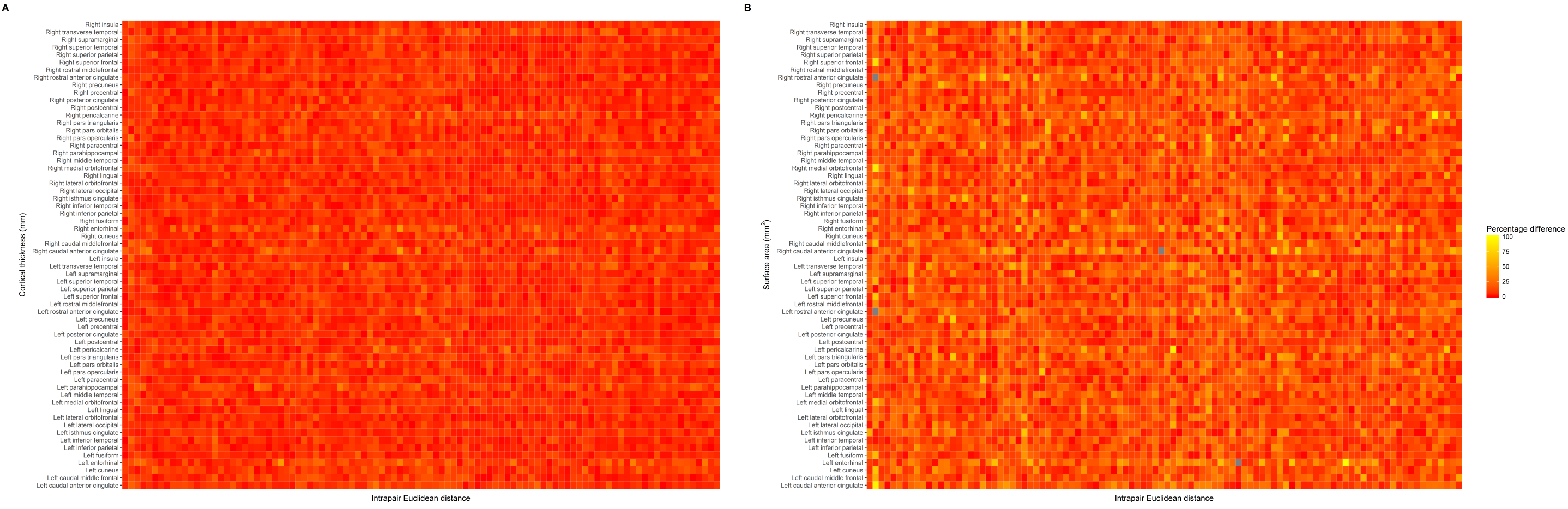

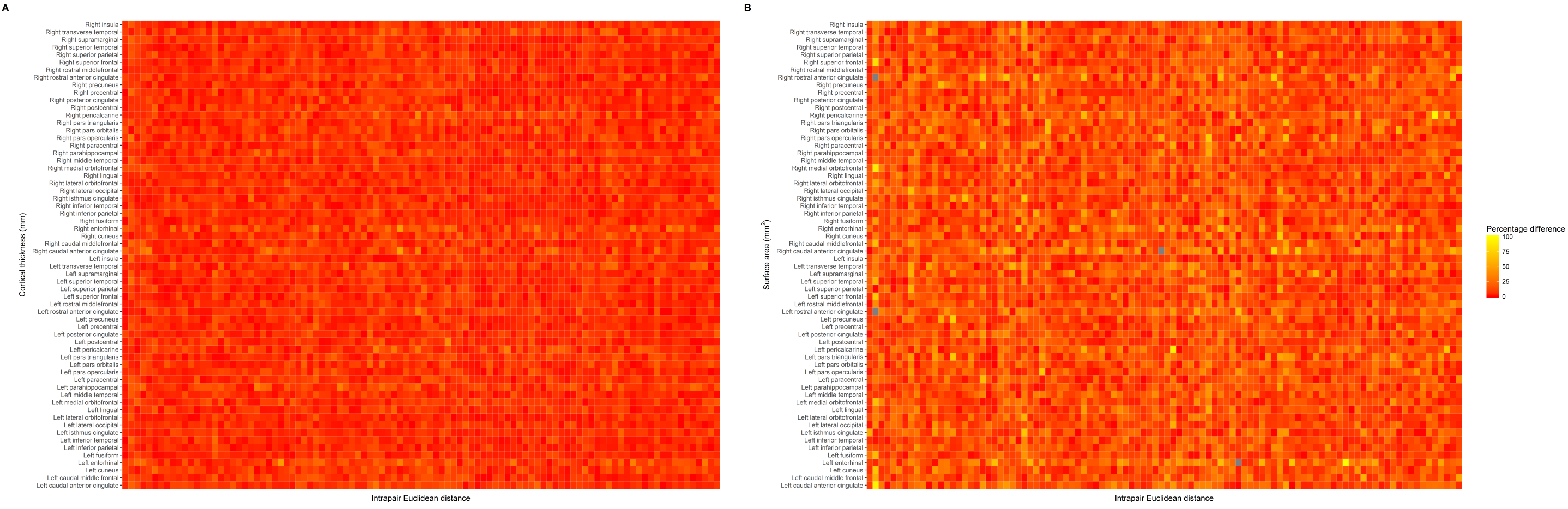


***Supplementary Figure 1. Exploratory data visualisation of intrapair differences for cortical thickness (A) and surface area (B).*** *Rows represents the within-pair percentage difference in each cortical region (columns). Brighter heatmap colours (yellow) indicates higher intrapair difference in structural morphometry features. Neutral regions (grey) indicate a percentage difference exceeding 100%.* *Neutral regions (grey) indicate a percentage difference exceeding 100%.* S*ubject-level differences in structural morphometry of specific regions, such as the anterior cingulate, appear to be higher than other cortical regions across most subjects.*

## Supplementary Figure 2. Out-of-sample predictions for cortical thickness and surface area features without subject-level modelling.


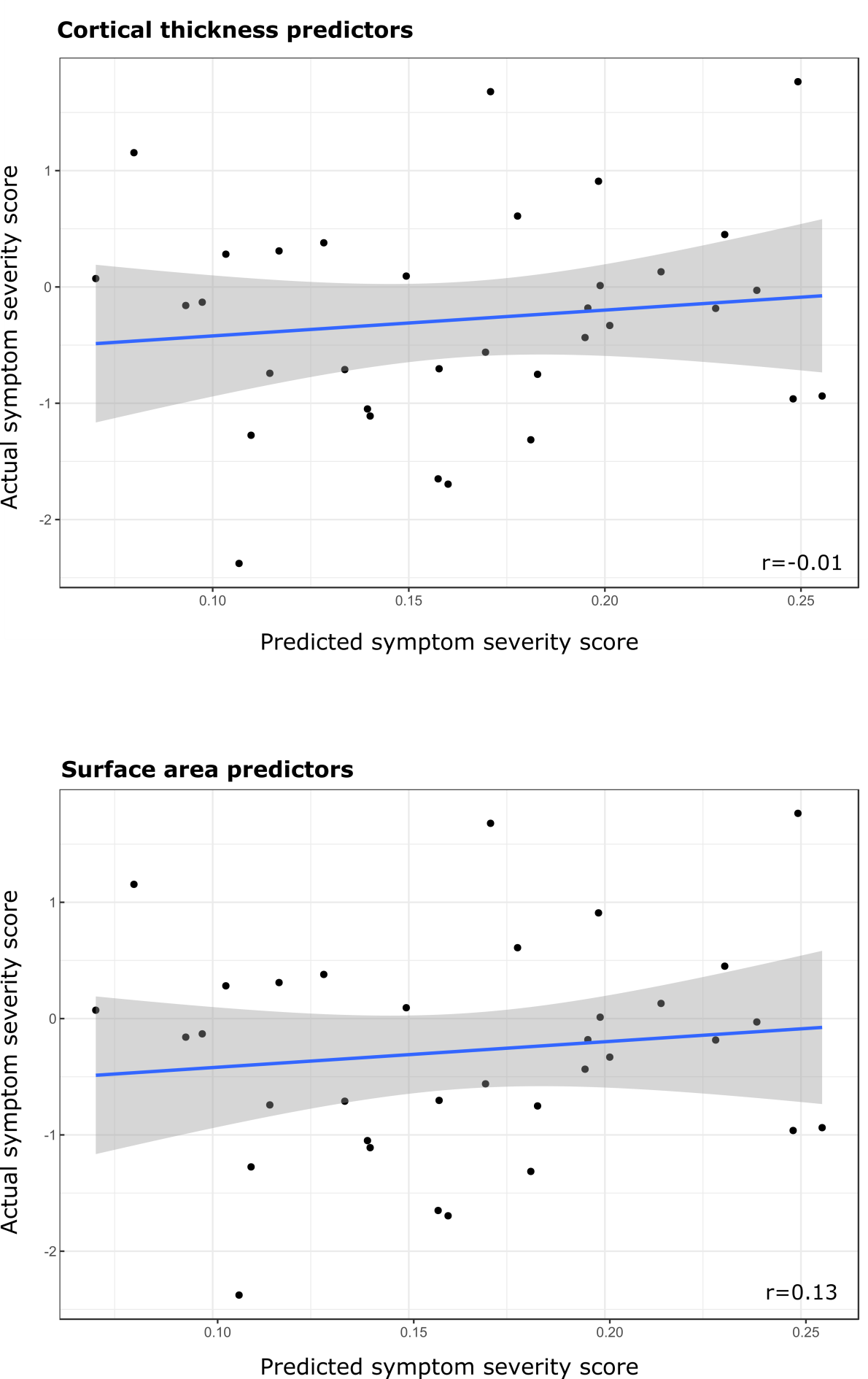


***Supplementary Figure 2. Out-of-sample predictions for cortical thickness and surface area features without subject-level modelling.*** *Regularized regression models for cortical thickness and surface area features trained on data without subject-level information performed poorly in predicting actual symptom severity scores in independent out-of-sample validation.*
